# Correction

**DOI:** 10.1080/13814788.2025.2477962

**Published:** 2025-04-01

**Article title:** Diverse roles of Primary Health Care in COVID-19 vaccination across 28 European countries – Insights from the Eurodata study

**Authors:**  Guisado-Clavero, M., Gómez-Bravo, R., Gefaell Larrondo, I. et al.

**Journal:** *European Journal of General Practice*

**Bibliometrics:** Volume 30, Issue 1 - 2024

**DOI:** https://doi.org/10.1080/13814788.2024.2409240

When this article was first published online, there was an error in the number of vaccines of Switzerland in Table 2. The line of Switzerland must be corrected to:

Total population (adults over 20 years): 6,995,806

Number of administered second dose: 5,410,092

Coverage of population with second dosage: 77.3%

As the authors correct the vaccination coverage, the color of Switzerland in Figure 1 (COVID-19 vaccination coverage of participant countries during the study period, full regime considered was 2 doses) must change to a lighter blue:

**Figure:**
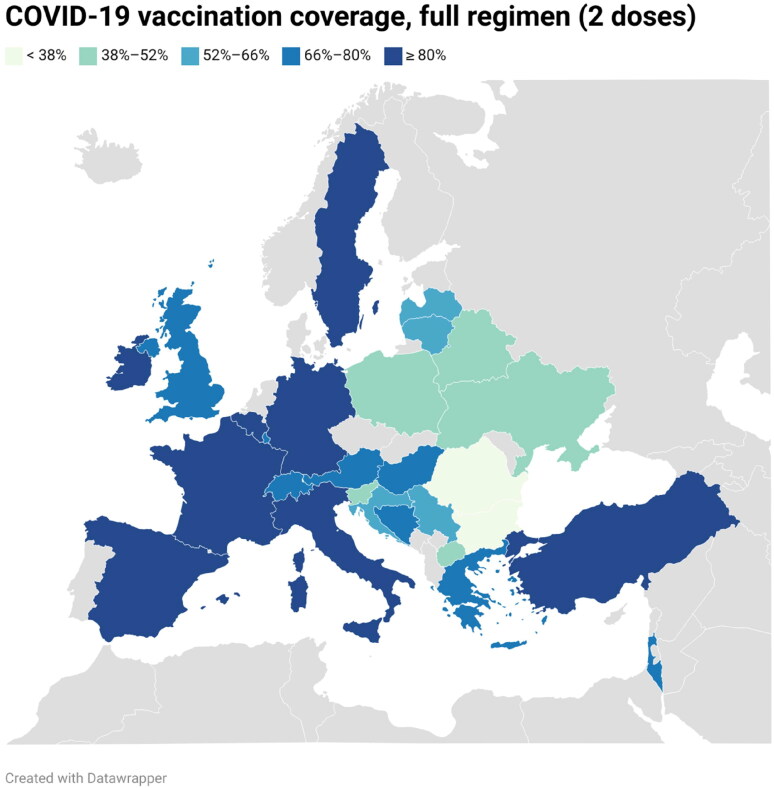


Footnote: Data from Switzerland has been collected from people over 20 years old.

In Supplement 2, an additional source should be added:

- Swiss open government data: https://opendata.swiss/en/dataset/covid-19-schweiz/resource/5c6f3227-41ab-4290-80e4-124791eef7c0

In the text: “In Germany, Greece, Hungary, Italy, and Ukraine, it was mandatory for certain groups (Table 3),…” ‘Table 3’ should be changed to ‘Supplement 3.’ Supplement 3 currently includes information on Germany, Poland, and Ukraine. Below are the details on the other countries where the COVID-19 vaccine was mandatory:

- **Hungary:** Healthcare workers in public institutions, staff of nursing homes and social care facilities, and elementary and middle school personnel.
- **Greece:** Healthcare workers, the elderly, individuals with multiple comorbidities, and residents of long-term care facilities.
- **Italy:** Healthcare and school personnel, firemen, police officers, and individuals over 50.

